# Competitive dCas9 binding as a mechanism for transcriptional control

**DOI:** 10.15252/msb.202110512

**Published:** 2021-11-08

**Authors:** Daniel A Anderson, Christopher A Voigt

**Affiliations:** ^1^ Synthetic Biology Center Department of Biological Engineering Massachusetts Institute of Technology Cambridge MA USA

**Keywords:** analog circuit, CRISPRi, ratio sensing, synthetic biology, Biotechnology & Synthetic Biology, Chromatin, Transcription & Genomics

## Abstract

Catalytically dead Cas9 (dCas9) is a programmable transcription factor that can be targeted to promoters through the design of small guide RNAs (sgRNAs), where it can function as an activator or repressor. Natural promoters use overlapping binding sites as a mechanism for signal integration, where the binding of one can block, displace, or augment the activity of the other. Here, we implemented this strategy in *Escherichia coli* using pairs of sgRNAs designed to repress and then derepress transcription through competitive binding. When designed to target a promoter, this led to 27‐fold repression and complete derepression. This system was also capable of ratiometric input comparison over two orders of magnitude. Additionally, we used this mechanism for promoter sequence‐independent control by adopting it for elongation control, achieving 8‐fold repression and 4‐fold derepression. This work demonstrates a new genetic control mechanism that could be used to build analog circuit or implement cis‐regulatory logic on CRISPRi‐targeted native genes.

## Graphical abstract

A regulatory control mechanism is developed based on the competitive binding of dCas9 to DNA by two guide RNAs. One represses a target gene (CRISPRi) and the second displaces the repressing dCas9 without interfering with the gene’s transcription.

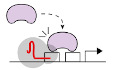

## Introduction

Regulatory networks integrate environmental and cellular signals to ensure genes are expressed under the correct conditions. Integration can occur at individual promoters through the arrangement of DNA operators to which regulatory proteins bind and either recruit or interfere with transcriptional machinery (van Hijum *et al*, 2009; Weingarten‐Gabbay & Segal, 2014). Within a promoter, the binding of one regulator can also affect the binding of others through positive or negative interactions, for example, through DNA looping or overlapping operators. This collectively generates “cis‐regulatory logic”, which dictates the conditions for gene expression based on the combination of regulators that are active (Shen‐Orr *et al*, 2002; Shen‐Orr *et al*, 2002; Aerts *et al*, 2003; Buchler *et al*, 2003; Hermsen *et al*, 2006; Mayo *et al*, 2006; Kaplan *et al*, 2008; Gertz *et al*, 2009; van Hijum *et al*, 2009; Schulthess *et al*, 2015). For synthetic genetic circuits, the placement of multiple operators within a promoter has been used to implement combinatorial logic, for example, to create an X AND (NOT Y) gate for an edge detector by placing an operator for an activator (X) and repressor (Y) within a promoter (Cox *et al*, 2007; Murphy *et al*, 2007; Ellis *et al*, 2009; Tabor *et al*, 2009; Sharon *et al*, 2012; Bacchus *et al*, 2013; Mogno *et al*, 2013; Perez‐Pinera *et al*, 2013; Rantasalo *et al*, 2018; Monteiro *et al*, 2020; Yu *et al*, 2021). As a design principle, combining operators is useful for “compressing” large logic operations to reduce the resource burden (Rondon *et al*, 2019; Groseclose *et al*, 2020). It is challenging to insert an operator into a promoter because the change in sequence can affect the promoter strength, an effect that can be mitigated with insulators and computational predictions (Brewster *et al*, 2012; Stanton *et al*, 2014; Zong *et al*, 2017, 2018; Hoque *et al*, 2021; preprint: Poole *et al*, 2021). Prokaryotic promoters are small, thus making it difficult to fit multiple operators and it is difficult or impossible to design operators that bind to multiple regulatory proteins.

dCas9 can be programmed to bind to different DNA sequences by changing the targeting sequence of its bound sgRNA (Bikard *et al*, 2013; Esvelt *et al*, 2013; Qi *et al*, 2013; Jiang & Doudna, 2017). DNA binding requires 5–10 bp sgRNA‐DNA complementarity adjacent to an NGG PAM sequence (Boyle *et al*, 2017), although dCas9 can be engineered to reduce the PAM sequence requirements (Kleinstiver *et al*, 2015; Hu *et al*, 2018; Nishimasu *et al*, 2018; Walton *et al*, 2020; Collias & Beisel, 2021). When bound to DNA, dCas9 covers 30 bp and melts the strands to form a bubble that results in very slow unbinding rates (Sternberg *et al*, 2014; Jones *et al*, 2017). Thus, it can function as a repressor by sterically blocking the binding of RNA polymerase (RNAP) to a promoter or by blocking its progression through a gene (Qi *et al*, 2013). When targeting constitutive *E. coli* σ70 promoters, repression is strongest when the sgRNA is targeted within the −10 to −35 promoter core and does not depend on which strand is targeted (Bikard *et al*, 2013; Qi *et al*, 2013; Nielsen & Voigt, 2014). Targeting dCas9 downstream of a promoter represses transcriptional elongation, with much stronger repression observed when the sgRNA is targeted to the non‐template strand (Bikard *et al*, 2013; Qi *et al*, 2013). dCas9 can also serve as an activator by fusing it to an activating domain that recruits RNAP (Bikard *et al*, 2013; Zalatan *et al*, 2015; Dong *et al*, 2018; Ho *et al*, 2020; Kiattisewee *et al*, 2021). There are often tight spacing requirements for activation, particularly for prokaryotic promoters. Lastly, dCas9 can also be directed to bind to the operators of protein repressors, activators, or enhancers, thus blocking their impact on expression (preprint: Shur & Murray, 2017; Shariati *et al*, 2019; Liu *et al*, 2021).

An advantage of using dCas9 is that its regulatory effect can be directed to a promoter without having to insert an operator sequence. This has been used to control the regulation of native genes, for example, controlling enzymes at branch points to redirect flux through a metabolic pathway (Kim *et al*, 2016; Moser *et al*, 2018; Tian *et al*, 2019; Hawkins *et al*, 2020; Wu *et al*, 2020b; van Gestel *et al*, 2021). It also simplifies the integration of multiple signals at a single promoter by designing sgRNAs that target it to different positions. For instance, NOR gates have been constructed using two sgRNAs that target different positions in the output promoter, either of which leads to repression (Lebar & Jerala, 2016; Gander *et al*, 2017). When multiple sgRNAs target overlapping regions, this leads to mutually exclusive binding (Qi *et al*, 2013). Tan and co‐workers harnessed this effect to control the strength and noise of an *E. coli* constitutive promoter by co‐transcribing different ratios of two sgRNAs that direct dCas9 (fused to an activating domain) to overlapping positions that either recruit or block RNAP (Wu *et al*, 2020a).

Once expressed, the regulatory effects of dCas9 only end when the protein or sgRNA degrade or are diluted by cell division. Several approaches have been taken to control the activity of either dCas9 or the sgRNAs after they are expressed. One is to express anti‐CRISPR proteins derived from phage genomes that bind to and inactivate dCas9 (Bubeck *et al*, 2018; Nakamura *et al*, 2019). This leads to the complete inactivation of dCas9, thus eliminating its ability to implement any sgRNA‐mediated regulation in the cell. Different sets of genes can be controlled by expressing orthogonal dCas9 variants, each of which binds a different set of sgRNAs (Gao *et al*, 2016; Kim *et al*, 2019). These can be changed dynamically from being repressors to activators by expressing the corresponding domains as separate proteins that bind to dCas9 using modular protein–protein interaction domains (Gao *et al*, 2016). Another approach is to design RNA to bind to and augment the activity of a specific sgRNA. Antisense RNAs will inactivate sgRNAs by targeting them for degradation via the native bacterial Hfq system (Lee *et al*, 2016). The sgRNA can also be designed to fold into an inactive hairpin, thus requiring the co‐expression of toehold RNAs to unfold and bind to dCas9 (Oesinghaus & Simmel, 2019; Siu & Chen, 2019; Hochrein *et al*, 2021). Both of these techniques require modifying the sgRNA to have additional sequences such that it can be bound by the modulating RNA.

**Figure 1:**
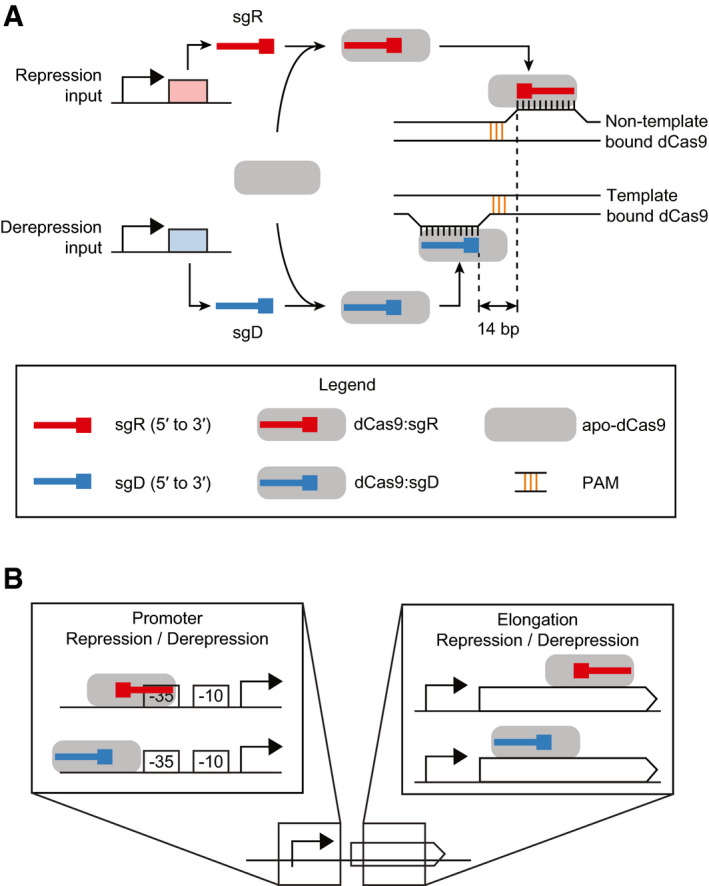
The mechanisms of dCas9 repression and derepression The footprint of dCas9:sgD blocks the binding of dCas9:sgR without requiring that the sgRNA‐binding regions overlap. The PAM‐adjacent regions of sgRNAs are shown as boxed ends.The sgR/sgD sequences can be targeted to the promoter or internal to a gene.

While there are many natural examples of different repressors binding to the same operator or a repressor displacing an activator, to our knowledge, there are no examples of a repressor’s action being negated by a second protein binding to an overlapping operator. To this end, we have developed a mechanism for sgRNA‐specific derepression through competitive dCas9 binding to overlapping regions. Transcription is blocked when a repressing sgRNA (sgR) directs dCas9 to the first position and a second derepressing sgRNA (sgD) directs dCas9 to a mutually exclusive second position that does not impact transcription (Fig 1A). We find that the two regions are competitive so long as they are within 14 bp and the PAM sequences are between the target sites. The repression/derepression switch is implemented in two ways (Fig 1B). The first is to design the sgR to overlap the −35 σ70‐binding region of promoter and the sgD to bind just upstream. The second approach is to design repression/derepression sites within a gene by exploiting the strand dependence of RNAP elongation inhibition by dCas9. These results demonstrate a new mechanism to control the activity of dCas9‐directed regulation that could be used for efficient genetic circuit design to integrate signals in a promoter or to derepress subsets of native genes to subregulate genome‐encoded functions.

## Results

### Promoter control through repression/derepression

**Figure 2:**
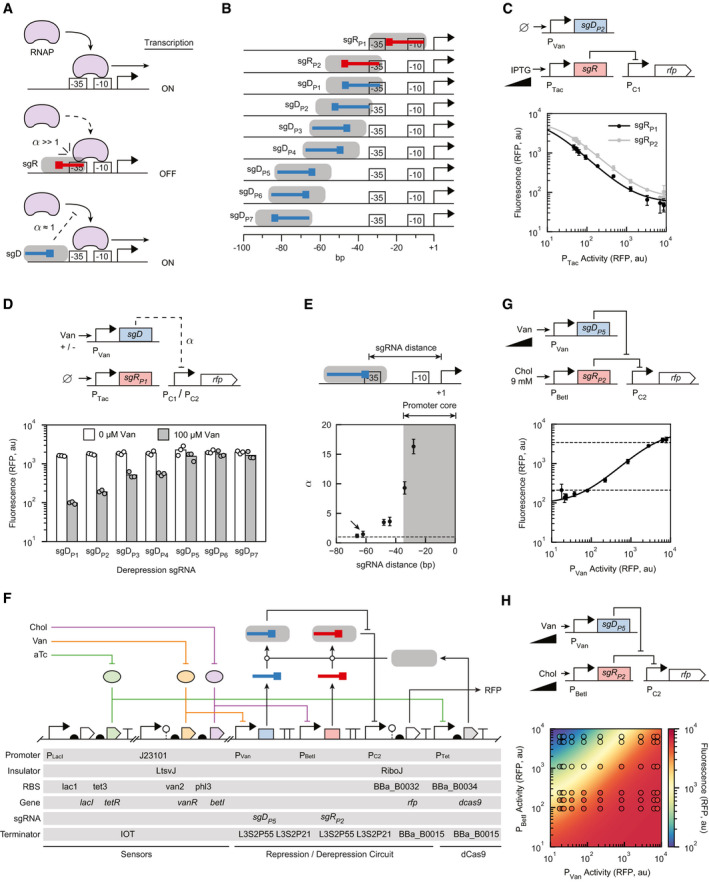
Repression and derepression targeted to a promoter The interactions between dCas9:sgR and dCas9:sgD and RNAP are shown. The dashed arrow and blunt‐end indicate RNAP’s inability to bind the promoter and non‐ideal repression of RNAP‐binding by a sgD, respectively.Positions of P_C1_ tested for targeting sgR and sgD. The boxes on the sgRNAs mark the PAM‐adjacent regions, the orientation of which determines which strand is targeted.Repression by the expression of sgRs. The experiments are performed with plasmids pDAA043 and pDAA656 for sgR_P1_ and sgR_P2_, respectively. Both plasmids contain sgD_P2_ (uninduced for these experiments). The curve was fit to Equation 4, yielding the parameters shown in Table 1. The *x*‐axis is converted from the concentration of inducer (IPTG) to promoter activity as described in the Materials and Methods. Inducer concentrations: 0, 0.977, 1.95, 3.91, 7.81, 15.6, 31.3, 62.5, 125, 250, 500, 1,000 µM IPTG.The position dependence of repression. The dashed blunt‐end arrow indicates non‐ideal repression by a derepression sgRNA. The experiments are performed with plasmids pDAA042, 043, 050, 051, 654, 052, and 044 for sgD_P1_‐sgD_P7_ (Appendix Table S1). The circuits also contain sgR_P1_ (uninduced for these experiments). The pDAA654 plasmid testing sgD_P5_ used the promoter P_C2_, all other sgDs used P_C1_.“sgRNA distance” is the distance from the +1 site of the promoter to the closest DNA–RNA base pair in the sgRNA sequence. The lower subpanel shows values of *α* for the sgDs tested in (D) as function of their distance from the +1 site. The horizontal dashed line is drawn at *α* = 1 (ideal *α* value). The arrow marks sgD_P5_, which was selected to build the repression/derepression circuit. The error bars were calculated as *σ* = |A/B|((*σ*_A_/A)^2^ + (*σ*_B_/B)^2^]^1/2^, where A and B are the means with 0 and 100 µM Van sgD induction, respectively, and *σ*_A_ and *σ*_B_ are the standard deviations of these measurements. Upper subpanel shows a schematic representation of how “sgRNA distance” values are calculated.Genetic circuit schematic of the circuit based on sgR_P2_ and sgD_P5_ circuit, encoded on a single plasmid (pDAA107). Part sequences are provided in Appendix Table S2, and the full plasmid sequence is in Appendix Table S3.Derepression induction curve for sgD_P5_ with full sgR_P2_ induction. The line is a fit to Equation 8 with parameters in Table 1. Dashed lines indicate RFP expression for uninduced (0 mM Chol, 0 µM Van) and fully induced (9 mM Chol, 0 µM Van) sgR_P5_. The *x*‐axis is converted from the concentration of inducer (Van) to promoter activity as described in the Materials and Methods. Inducer concentrations: 0, 0.0977, 0.195, 0.391, 0.781, 1.56, 3.13, 6.25, 12.5, 25, 50, 100 µM. The upper subpanel is a genetic circuit schematic of the sgR_P2_ + sgD_P5_ circuit (full schematic shown in F).Two‐dimensional response function for the induction of sgR_P2_ and sgD_P5_. The circles are experimental measurements colored by the mean fluorescence values of three biological replicates performed on different days (standard deviations in Appendix Fig S4). The continuous color in the background is the model prediction from Equation 8, *R* ^2^ = 0.87 (Appendix Fig S4). Inducer concentrations: [0, 0.195, 0.391, 0.781, 1.56, 3.13, 6.25, 12.5, 25, 50, 100 µM Van] and [0, 37.0, 111, 333, 1,000, 3,000, 9,000 µM Chol]. 27‐fold repression was observed with 0.195 µM Van and 0 / 9 mM Chol applied. 29.5‐fold derepression was observed with 9 mM Chol and 0.195 / 100 µM Van applied. Data information: Representative cytometry distributions for all parts are shown in Appendix Fig S3. For panels (C), (E), (G), and (H), data points are the means of three biological replicates performed on different days. For panel (D), all data points are shown. In panels (C), (E), and (G), the error bars are the standard deviations of these measurements.

dCas9 can repress a promoter by sterically blocking the binding of RNAP (Figs 1B and 2A) (Bikard *et al*, 2013; Qi *et al*, 2013). An *E. coli* constitutive promoter can be repressed by targeting the repressing sgRNA (sgR) to overlap the −10 or −35 σ70‐binding sites (Bikard *et al*, 2013; Qi *et al*, 2013; Nielsen & Voigt, 2014). Our design for derepression is based on using a second sgRNA (sgD) to recruit dCas9 to an upstream site that blocks its ability to bind to the repressing position. Importantly, sgD cannot interfere with RNAP binding or else it will also lead to repression. To quantify the impact of dCas9:sgRNA binding on mRNA transcription rates, we defined a parameter α as the ratio of maximal mRNA production rates (completely unbound DNA) to the production rate when all DNA is bound by dCas9:sgRNA (saturated DNA). The ideal location to target a repression/derepression sgRNA pair would correspond to a large α for sgR (α >> 1) and α → 1 for sgD, indicating strong repression by sgR and no repression by sgD.

A system was designed to evaluate how targeting sgR and sgD to different positions in the promoter impacts the effectiveness of repression and derepression. Two inducible promoters were used to independently transcribe sgR (IPTG‐inducible P_Tac_) and sgD (Vanillic acid‐inducible P_Van_) encoded on a p15A plasmid. The targeted constitutive promoter (P_C1_) was built based on the −35 to +1 core of P_Gate08_ (Zhang & Voigt, 2018) to which a randomly generated 100‐bp sequence was added upstream (Materials and Methods). Positions in P_C1_ were selected to be targeted for sgR/sgD by exploiting NGG PAM sites in the promoter sequence (Fig 2B). The sgR and sgD sequences were designed based on the same scaffold (Zhang & Voigt, 2018), with mutations the 20‐bp spacer sequence corresponding to the target region of the promoter (Appendix Table S2). To measure promoter activity, P_C1_ was placed upstream of a ribozyme (Lou *et al*, 2012) and gene encoding red fluorescent protein (*rfp*). From the same plasmid, dCas9 was expressed using the aTc‐inducible P_Tet_ promoter. A concentration of 1.25 nM aTc was used for all experiments and leads to approximately 500 dCas9 molecules per cell during exponential growth (Zhang & Voigt, 2018).

Experiments were performed to measure the promoter activity that results when dCas9 is targeted to different positions. The plasmids were transformed into *E. coli* and cultures were grown in defined media (EZ Rich) and induced with aTc and either IPTG or Vanillic acid (Van) for 5.5 h (Materials and Methods). Fluorescence was then measured using flow cytometry. First, we compared the repression obtained by targeting the −10 (sgR_P1_) or −35 (sgR_P2_) positions of the promoter (Fig 2B). Upon maximum sgR expression (1 mM IPTG), both sgRs are able to repress the promoter by ˜ 30‐fold (Fig 2C). This result is consistent with previously observed fold‐repressions at these locations (Nielsen & Voigt, 2014; Zhang & Voigt, 2018).

A model was derived to capture the repression of a promoter by dCas9:sgR,

(1) $$P+S_{R}\overset{K_{R}}{\Leftrightarrow }P_{R}$$

where *P* and *P_R_* are the concentrations of promoters in the unbound and bound state, *S_R_* is the concentration of dCas9:sgR, and *K_R_* is the association constant. The production of mRNA transcripts *m* from the promoter is described by

(2) $$\frac{\mathrm{dm}}{\mathrm{dt}}\;=\;\beta_{m}\frac{1\;+\alpha^{‐1}K_{R}S_{R}}{1+K_{R}S_{R}}\;+\;\beta ‐\delta_{m}m,$$

where *β*_m_ is the maximum transcription rate, *β* is the leaky transcription rate, and *δ_m_* is the degradation rate. Solving for steady‐state yields

(3) $$m=\left(\frac{\beta_{m}}{\delta_{m}}\right)\frac{1+\alpha^{‐1}K_{R}S_{R}}{1+K_{R}S_{R}}\;+\;\frac{\beta }{\delta_{m}}.$$

This equation can be further converted to the activity of the output promoter P_C1_,

(4) $$y=\left(y_{\max}‐y_{\min}\right)\frac{1+\alpha^{‐1}\kappa_{R}x_{R}}{1+\kappa_{R}x_{R}}\;+\;y_{\min}$$

where *y* is in arbitrary units (au) of RFP fluorescence and *y*_max_/*y*_min_ are the maximum/minimum measured values. *x_R_* is the strength of the promoter driving the expression of sgR, measured as au of RFP fluorescence and *κ_R_* is rescaled to be in the same units. This assumes that the binding of sgR to dCas9 to form *S_R_* is in the linear (unsaturated) regime. Equation 4 was fit to the sgR_P1_ and sgR_P2_ induction curves (Fig 2C), and the parameters were extracted (Table 1).

**Table 1:** Model parameters.

		Parameters					
Mechanism	sgRNAs^a^	*y*_min_	*y*_max_	*κ_R_*	*κ_D_*	*α_R_*	*α_D_*
Promoter	sgR_P1_	1.2	7,700	0.10		150	
Promoter	sgR_P2_	16	6,800	0.043		120	
Promoter	sgR_P2_+sgD_P5_	16	6,800	0.043	0.063	120	1.0
Elongation	sgR_O1_	37	4,200	0.019		27	
Elongation	sgR_O1_+sgD_O2_	37	4,200	0.019	0.0077	27	1.0

^a^ Single sgRNA experiments were fit to Equation 4 and dual‐sgRNA experiments were fit to Equation 8.

We then designed experiments to determine the constraints of targeting dCas9:sgD to the promoter without evoking repression (Fig 2D). Seven positions were selected between −28 and −66 regions of the promoter, including orientations that target both strands (Fig 2B). The promoter activity was measured for each position when sgD is expressed (100 µM Van) and unexpressed (0 µM Van), and these data were used to estimate α as the ratio of these values (Fig 2D and E). We observed that α continuously decreases as the sgD targets regions farther from the promoter core, indicating weaker repressive abilities (Fig 2E). When sgD targets regions upstream of −60, negligible repression was observed.

It has been previously shown that dCas9:sgRNAs will compete for binding when their target regions overlap (Qi *et al*, 2013; Wu *et al*, 2020a). However, the distance constraints we measured showed that it would not be possible to obtain repression/derepression by targeting overlapping regions. Even if we used the upper‐bound on TSS distance for the repressing sgRNA (30 bp) and the lower‐bound on distance for the derepressing sgRNA (60 bp), the sgRNA target sequences would still have to be 10 bp away from each other. We hypothesized that while we could not utilize sgRNA target overlap, we may still be able to harness steric hindrance effects from overlap of the dCas9 protein footprint on DNA. To investigate this, we examined a crystal structure of the dCas9‐sgRNA‐DNA complex (Nishimasu *et al*, 2014), which shows that dCas9 has an overhang of ˜ 9 bp on the PAM‐proximal binding side, but only a ˜ 1 bp overhang on the PAM‐distal side of the sgRNA target. This indicated that we might be able to obtain competitive dCas9 binding without overlapping the sgRNA binding regions if we oriented two sgRNAs with their PAM sequences facing each other.

To test this hypothesis, we redesigned the system to express sgR_P2_ from a choline (Chol)‐inducible promoter and sgD_P5_ from a Van‐inducible promoter (Fig 2F). These promoters were chosen because they are not predicted to append disruptive 5′ sequences onto the sgRNAs (Qi *et al*, 2013; Meyer *et al*, 2019). In accordance with the crystal structure estimation of dCas9’s DNA footprint, we designed sgD_P5_ to be 14 bp from sgR_P2_. To bind sgD_P5_ at this distance, we had to introduce a 3‐bp mutation from −60 to −62 of P_C1_ to create a PAM site for sgD_P5_, resulting in P_C2_. We screened for derepression by fully inducing sgR_P2_ with 9 mM Chol and titrating the expression of sgD_P5_ from 0 to 100 µM Van. Derepression occurred in a graded manner as more sgRNA is expressed, ultimately returning the promoter activity to its unrepressed state (Fig 2G).

We then determined the promoter response when different amounts of sgR and sgD are expressed. This response can be viewed as a cis‐regulatory logic operation (Mayo *et al*, 2006), where the signals from these regulators are integrated by the promoter. To obtain this function, 60 combinations of sgR_P2_ and sgD_P5_ induction levels were measured. These data are shown in Fig 2H as circles, where their colors are the output values of the circuit and each data point is positioned at the respective promoter activity values for sgR and sgD (error bars for each measurement are provided in Appendix Fig S4). Output from the promoter increased with sgD induction and decreased with sgR expression.

It has been shown that the co‐expression of multiple sgRNAs titrates a shared dCas9 resource away from the co‐expressed sgRNAs (Zhang & Voigt, 2018; Huang *et al*, 2021). This could complicate the interpretation of the derepression data, where the expression of sgD could deplete the dCas9 pool, thus indirectly reducing the concentration of dCas9:sgR in the cell. To test for this effect, we performed a control experiment where we maximally expressed a sgR and then titrated in either a non‐targeting sgRNA (sgN) (does not bind to the promoter or genome) or an off‐target sgRNA (sgO) (binds to an inert region of the plasmid) (Appendix Fig S2). Neither of these sgRNAs showed derepression, indicating that the derepression we observed was not due to dCas9 titration.

Our model was then expanded to include derepression. Equation 1 can be modified to include the competitive reaction of dCas9:sgD (*S_D_*) binding to the promoter to form *P_D_*,

(5) $$P_{D}+S_{R}\overset{K_{D}}{\Leftrightarrow }P+S_{R}+S_{D}\overset{K_{R}}{\Leftrightarrow }P_{R}+S_{D}$$

where *K_D_* is the association constant between *S_D_* and *P*. The production of mRNA transcripts *m* from the promoter is described by

(6) $$\frac{\mathrm{dm}}{\mathrm{dt}}=\beta_{m}\frac{1+\alpha_{R}^{‐1}K_{R}S_{R}+\alpha_{D}^{‐1}K_{D}S_{D}}{1+K_{R}S_{R}+K_{D}S_{D}}\;+\;\beta ‐\delta_{m}m$$

where *β_m_*/*α_R_* and *β_m_*/*α_D_* are the maximum transcription rates when either *S_R_* or *S_D_* are bound to the promoter. Solving for steady‐state yields

(7) $$m=\left(\frac{\beta_{m}}{\delta_{m}}\right)\frac{1+\alpha_{R}^{‐1}K_{R}S_{R}+\alpha_{D}^{‐1}K_{D}S_{D}}{1+K_{R}S_{R}+K_{D}S_{D}}\;+\;\frac{\beta }{\delta_{m}}$$

As described for Equation 4, this can be further converted to the activity of the output promoter P_C2_,

(8) $$y=\left(y_{\max}‐y_{\min}\right)\frac{1+\alpha_{R}^{‐1}\kappa_{R}x_{R}+\alpha_{D}^{‐1}\kappa_{D}x_{D}}{1+\kappa_{R}x_{R}+\kappa_{D}x_{D}}\;+\;y_{\min}$$

This equation was then fit to the 2‐dimensional response of the promoter to the expression of sgR and sgD (Fig 2H). This fit was performed while keeping the parameters previously fit to Equation 4 for sgR_P2_ (*κ_R_*, *α_R_, y*_min_, and *y*_max_) constant. The fit is shown as the heatmap coloration in Fig 2H, the parameters for which are provided in Table 1. The newly fit association constant *κ_D_* for sgD is similar to *κ_R_* indicating that sgR_P2_ and sgD_P5_ have similar apparent binding strengths. Additionally, the observed α_D_ of 1 is consistent with sgD_P5_ having little repressive effect on its own.

### Dynamics of derepression

Qi *et al* (2013) showed that, in exponentially growing *E. coli*, the dynamics of sgRNA‐based repression of RFP was identical to the cellular growth rate. This is consistent with complete repression of protein expression and first‐order degradation of the protein due to dilution by cell division (Del Vecchio & Murray, 2015; Potvin‐Trottier *et al*, 2016). However, when the authors removed the repressing sgRNA, RFP fluorescence increased with a doubling time slower than the cellular growth rate.

**Figure 3:**
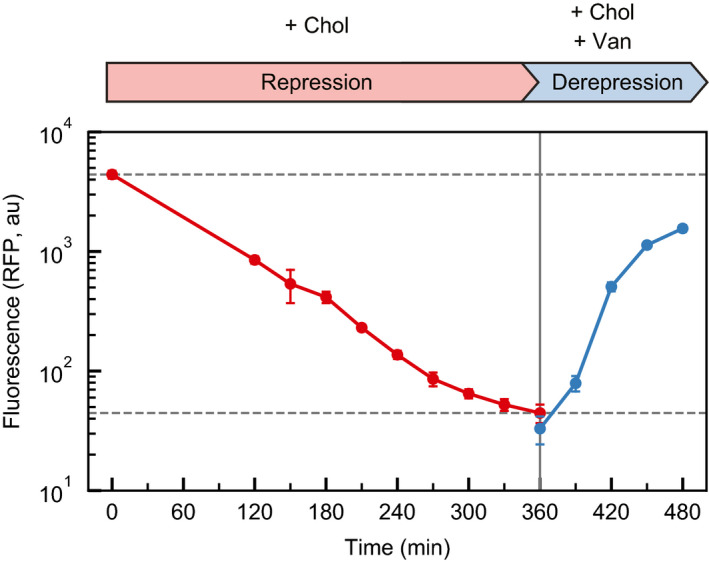
Dynamics of repression and derepression The mean fluorescence is shown after inducer is added for the expression of sgR (red points) and then expression of both sgR and sgD (blue points). The dashed lines mark maximum and minimum fluorescence observed during sgR repression. A vertical line indicates the time when cells were washed and transferred to derepressing conditions (Materials and Methods). Each data point represents the mean of four biological replicates performed over 2 days and the error bars are the standard deviations of these measurements. Lines connecting data points are drawn as a visual aid.

We monitored the dynamics of repression and derepression for the promoter repression/derepression sgRNA pair sgR_P2_ + sgD_P5_ over the course of 8 h (Materials and Methods). Starting with uninduced cells in exponential phase, we diluted cells into repressing conditions (10 mM Chol, 1.25 nM aTc). Consistent with previous work, RFP fluorescence reduced at the rate of cellular division (Fig 3). After 6 h, RFP expression was 100‐fold lower than the initial uninduced condition. We then washed the cells and diluted them into fresh media inducing only the derepression sgRNA, sgD_P5_ (1.25 nM aTc and 100 µM Van). This led to 47‐fold derepression within 2 h.

### Control of transcriptional elongation through repression/derepression

**Figure 4:**
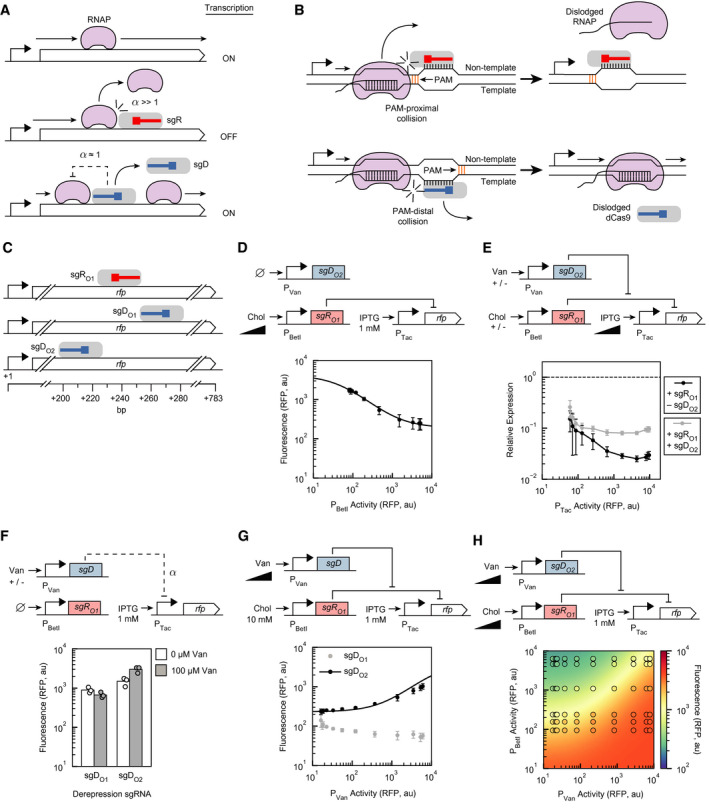
Repression/derepression by blocking RNAP progression within a gene The interaction between RNAP and dCas9:sgR and dCas9:sgD are shown. Transcription is “ON” when full‐length transcripts are produced. The dashed lines indicate non‐ideal repression by sgD.When dCas9 is bound to the non‐template strand, the PAM is proximal to RNAP, and dCas9 tends to stay bound after collision. When dCas9 is bound to the template strand, the PAM is distal to RNAP collision and RNAP can continue to elongate.Repressing and derepressing sgRNA binding sites tested relative to *rfp*.Repression of transcriptional elongation by sgR_O1_. The circuit is encoded on plasmid pDAA056, which also includes sgD_O2_ (uninduced in these experiments). The curve was fit to Equation 4, yielding the parameters shown in Table 1. The *x*‐axis is converted from the concentration of inducer (Chol) to promoter activity as described in the Materials and Methods. Inducer concentrations: 0, 9.77, 19.5, 39.1, 78.1, 156, 313, 625, 1,250, 2,500, 5,000, 10,000 µM Chol.The dependence of sgR_O1_ repression and sgD_O2_ derepression on the strength of the promoter controlling the repressed gene (P_Tac_). “Relative expression” was calculated as the ratio of the fluorescence obtained in the absence of a guide RNA (pDAA040) by the fluorescence obtained by the expression of the guide RNAs shown in the legend (10 mM Chol or 10 mM Chol/100 µM Van, using pDAA056). The lines are a visual guide. The *x*‐axis is converted from the concentration of inducer (IPTG) to promoter activity as described in the Materials and Methods. Inducer concentrations: 0, 0.977, 1.95, 3.91, 7.81, 15.6, 31.3, 62.5, 125, 250, 500, 1,000 µM IPTG. The error bars were calculated as *σ* = |A/B|((*σ*_A_/A)^2^ + (*σ*_B_/B)^2^]^1/2^, where A is the mean of the P_Tac_‐only expression, B is the mean of the circuit with either sgR_O1_ or sgR_O1_ + sgD_O2_ induction, and *σ*_A_ and *σ*_B_ are the standard deviations of their measurements.Fully induced P_Tac_ activity with and without induction of sgDs. Plasmids pDAA057 and pDAA056 were used for the sgD_O1_ and sgD_O2_ experiments, respectively. sgR_O1_ was present but uninduced.Derepression induction curves with full sgR_O1_ induction. The curve was fit to Equation 8, yielding the parameters shown in Table 1. The *x*‐axis is converted from the concentration of inducer (Van) to promoter activity as described in the Materials and Methods. Inducer concentrations: 0, 0.0977, 0.195, 0.391, 0.781, 1.56, 3.13, 6.25, 12.5, 25, 50, 100 µM Van.Two‐dimensional response function for the induction of sgR_O1_ and sgD_O2_. The circles are experimental measurements colored by the mean fluorescence values of three biological replicates performed on different days (standard deviations in Appendix Fig S4). The continuous color in the background is the model prediction using Equation 8, *R* ^2^ = 0.87 (Appendix Fig S4). The *x*‐axis and *y*‐axis are converted from Van and Chol to promoter activity, respectively (Materials and Methods). Inducer concentrations: [0, 0.195, 0.391, 0.781, 1.56, 3.13, 6.25, 12.5, 25, 50, 100 µM Van] and [0, 37.0, 111, 333, 1,000, 3,000, 9,000 µM Chol]. 8‐fold repression was observed with 0.195 µM Van and 0 / 9 mM Chol applied. 4‐fold derepression was observed with 9 mM Chol and 0.195 / 100 µM Van applied. Data information: Representative cytometry distributions for all parts are shown in Appendix Fig S3. For panels (D) and (G), data points are the means of three biological replicates performed on different days. For panel (F), all data points are shown. In panels (D) and (E), the error bars are the standard deviations of the three separate measurements.

dCas9 can block transcription when it is directed to bind internally to a gene by physically interfering with the progression of RNAP (Fig 4A). However, being located in a gene complicates the design of a derepressing sgRNA position, which must bind to disrupt the repressing sgRNA without itself blocking elongation. To this end, we exploited the strand dependence of dCas9‐based elongation repression, where it has been found that RNAP elongation is more likely to terminate when it collides with the PAM‐proximal side of a dCas9‐DNA complex (Fig 4B) (Bikard *et al*, 2013; Qi *et al*, 2013; Vigouroux *et al*, 2018). Similar to the promoter repression/derepression mechanism, these constraints on sgR and sgD repressive effects can be captured empirically with the parameter α, which is the maximum fold‐repression of mRNA production rates under saturating conditions.

A genetic system was constructed to test this design. dCas9 produces stronger repression when it is directed to the 5’‐end of the gene (Bikard *et al*, 2013; Qi *et al*, 2013), so we selected a position at +232 to be targeted by a repressing sgRNA (sgR_O1_) (Fig 4C). This location was chosen because it has two adjacent PAM sites that could be used for targeting sgD, one of which has the same 14 bp spacing as was found to be optimal when derepressing a promoter (sgD_O2_). sgR_O1_ was placed under the control of a Chol‐inducible P_BetI_ promoter. It has been observed that elongation repression can be weaker with stronger promoters, presumably due to dislodgement of dCas9 by RNAP (Vigouroux *et al*, 2018). To evaluate this effect, we used an IPTG‐inducible promoter (P_Tac_) to drive the transcription of the *rfp* reporter. When the promoter is strong (1 mM IPTG), we observed an 8‐fold repression of RFP upon the maximum induction of sgR_O1_ (10 mM Chol) (Fig 4D). While we initially developed the models in Equations 4 and 9 for the promoter repression/derepression mechanism, these models can be generalized to the elongation repression/derepression mechanism if the promoter states *P*, *P_R_*, and *P_D_* are instead considered to be general DNA states. Therefore, the data for sgR_O1_ were fit to Equation 4 (Table 1). The α for sgR_O1_ is 4‐fold lower than that from promoter repression (Table 1), indicating a limitation of elongation repressive control at this location. The dependence of repression on the P_Tac_ activity was then measured (Fig 4E). Repression by sgR_O1_ initially increased with promoter strength, but then levels off at 30‐fold repression (Fig 4E). Previous work showed that elongation repression is promoter‐strength invariant for saturating levels of dCas9 and for strong promoters (Vigouroux *et al*, 2018).

Two sgDs were designed to target positions up‐ (sgD_O2_) and down‐stream (sgD_O1_) of the sgR_O1_ position (Fig 4C). These were placed under the control of a Van‐inducible promoter, as before. We initially attempted to calculate α for these derepressing sgRNAs; however, induction of sgD_O2_ resulted in higher, rather than lower, RFP expression. This effect was likely due to leaky sgR_O1_ expression of sgR_O1_ from P_BetI_ which is derepressed when sgD_O2_ is induced (Fig 4F). Nonetheless, we tested the derepression capabilities of sgD_O1_ and sgD_O2_ (Fig 4G). To do this, sgR_O1_ was maximally expressed (10 mM Chol) with the strongest induction of the *rfp* reporter (1 mM IPTG). Maximum expression of sgD_O2_ (100 µM Van) showed 4‐fold derepression (Fig 4G). This derepression was confirmed to not be a result of the titration of dCas9 (Appendix Fig S2). In contrast, sgD_O1_ did not show any derepression and instead its expression slightly reduces the expression of the reporter, implying it may have its own repressing effect.

The regulatory logic for the co‐expression of sgR_O1_ and sgD_O2_ was then determined (Fig 4H). Similar to promoter repression/derepression, the elongation mechanism exhibited increasing expression with sgD induction and decreasing expression with sgR induction (error bars for each measurement are provided in Appendix Fig S4). Equation 8 was then fit to the 60‐point sampling of the system, with *x_R_* corresponding to P_BetI_ activity and *x_D_* corresponding to P_Van_ activity values. The fit parameters imply that the derepressing sgRNA association constant *κ_D_* was 2.5‐fold lower than the repressing sgRNA association constant *κ_R_* (Table 1).

The dependence of derepression on the P_Tac_ promoter strength was then measured by varying IPTG (Fig 4E). Similar to the promoter strength test for relative repression, we normalized the fluorescence to that of the P_Tac_‐only plasmid (pDAA040). For each level of P_Tac_ induction, the relative expression during derepression was calculated as the ratio of the RFP fluorescence from the P_Tac_‐only plasmid to the RFP fluorescence from the circuit plasmid with maximal sgR_O1_ and sgD_O2_ induction (10 mM Chol and 100 µM Van). From this experiment, we observed that derepression is invariant over a wider range of transcription rates than that observed for repression.

### Ratiometric performance of the repression/derepression

**Figure 5:**
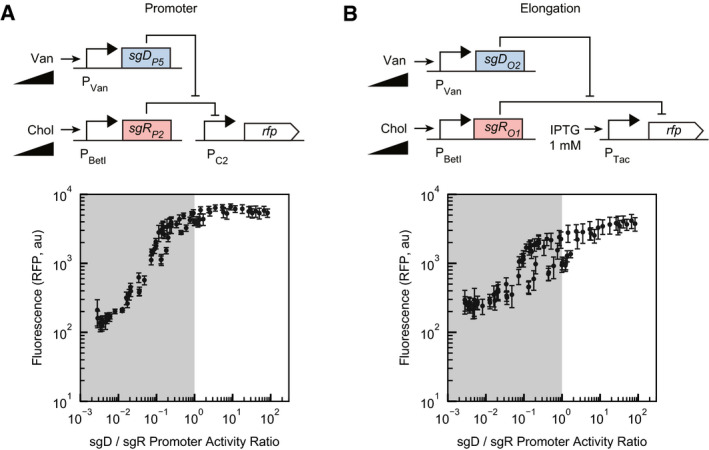
Ratiometric performance of repression/derepression circuits A, B(A) Promoter repression/derepression circuit experimental results with a ratiometric interpretation. The upper subpanel is a genetic circuit schematic of the sgR_P2_ + sgD_P5_ circuit (full schematic shown in Fig 2F). Lower subpanel is the data from Fig 2H replotted as the ratio of the two inputs. The *x*‐axis values are derived by taking the ratio of P_Van_ activity to P_BetI_ activity. (B) The upper subpanel is a genetic circuit schematic of the sgD_O2_ + sgR_O1_ circuit. The lower subpanel is the elongation repression/derepression circuit data (Fig 4H) replotted as the ratio of the two inputs. For both parts, the *x*‐axis values are derived from converting Chol (sgR) and Van (sgD) values to promoter activity (Materials and Methods) and then dividing sgD promoter activity by sgR promoter activity. Inducer concentrations: [0, 0.195, 0.391, 0.781, 1.56, 3.13, 6.25, 12.5, 25, 50, 100 µM Van] and [0, 37.0, 111, 333, 1,000, 3,000, 9,000 µM Chol]. Ratios below 1 are shown in gray. For both panels, data points are the means of three biological replicates performed on different days and the error bars are the standard deviations of these measurements.

Ratiometric signal processing describes a circuit that responds to the relative value of two inputs, as opposed to their absolute magnitude. Naturally occurring ratiometric responses have been observed in ATP/ADP management (Atkinson, 1968), X versus autosomal chromosome levels (Madl & Herman, 1979), circadian clock determination (Li *et al*, 2016), cancer cell clinical resistance (Raisova *et al*, 2001), and sugar source utilization in yeast (Escalante‐Chong *et al*, 2015). To examine the ratiometric performance of our repression/derepression mechanisms, we looked at the circuit outputs relative to the ratio of promoter activities producing sgD and sgR (P_Van_ / P_BetI_). From this, we observed that the promoter repression/derepression circuit effectively responded to over 2 orders of magnitude of sgD/sgR ratios with a 50‐fold dynamic range (Fig 5A). In contrast, the elongation mechanism had a smaller overall dynamic range (10‐fold) and the circuit did not perform predictably across all ratios (Fig 5B).

## Discussion

This work introduces a new mode of regulatory control, where the binding of a repressor is displaced by the binding of a nearby derepressor. This motif, where both repressor and derepressor bind to the same operator, has not been observed in natural systems. This may be due to the difficulty of designing a common operator that binds to multiple proteins and a derepressing protein that blocks the repressor, but not RNAP. The programmability of CRISPRi makes the design of multiple sgRNAs binding nearby sites almost trivial and the size of dCas9, and its melting of DNA, simplifies its use to sterically inhibit the binding or elongation of RNAP. Note that the off‐rate of dCas9 binding DNA is very slow, effectively irreversible, until it is displaced by DNAP or RNAP (Sternberg *et al*, 2014; Jones *et al*, 2017; Vigouroux *et al*, 2018). In our repression/derepression mechanism, this makes it unlikely that dCas9:sgD can displace dCas9:sgR when it is already bound. Rather, it depends on which binds first, with DNAP replication (and to a lesser‐extent RNAP elongation) “resetting” the DNA state at the rate of plasmid replication (cellular growth rate) and RNAP elongation (promoter strength) (Jones *et al*, 2017; Vigouroux *et al*, 2018). Thus, the kinetics of the system reaching steady state are expected to be dependent on growth phase and may impact their use in circuits with components with faster dynamics (Mishra *et al*, 2014; Takahashi *et al*, 2015; Ali Al‐Radhawi *et al*, 2019; Westbrook *et al*, 2019).

Repression/derepression could be applied to design synthetic circuits that perform new signal integration functions. As simple cis‐regulatory logic, the operation performed by these circuits is A IMPLY B where sgD (B) overrides the regulation imposed by sgR (A) (Figs 2G and 3G). If instead the displaced regulator were an activator (based on dCas9 recruiting RNAP (Dong *et al*, 2018; Fontana *et al*, 2020a; Ho *et al*, 2020; Villegas Kcam *et al*, 2021)), this logic would be A NIMPLY B.

However, this digital combinatorial logic does not capture the potential for new signal integration performed by these mechanisms. The signal integration follows a graded, rather than switch‐like transition (Figs 2H and 3H), and this can be used to build useful analog functions. The circuit based on the repression/derepression of a promoter (sgR_P2_ and sgD_P5_) is capable of responding to transcriptional input ratios over two orders of magnitude (Fig 5A). Ratiometric signal processing is common in natural regulatory systems and is usually achieved by a motif where two species compete for a third component and only one of the bound species results in the output (Atkinson, 1968; Madl & Herman, 1979; Raisova *et al*, 2001; Berg *et al*, 2009; Daniel *et al*, 2013; Escalante‐Chong *et al*, 2015; Li *et al*, 2016; Antebi *et al*, 2017; Cherry & Qian, 2018; Lopez *et al*, 2018; Liu *et al*, 2019). For example, this competition can occur at a DNA binding site where an activating transcription factor competes with an inactive or repressive transcription factor (Perli & Lu, 2017; Zeng *et al*, 2019). Ratiometric circuits can be used to determine which of two continuously variable signals is larger. The sgR_P2_/sgD_P5_ circuit in log‐space is performing a (sgD – sgR) calculation, which if connected to a cooperative switch‐like output could act as a single neuron in a neural network (Li *et al*, 2021).

Another consideration is the transfer of this repression/derepression mechanism for transcriptional control in organisms other than *E. coli*. Repression of transcriptional elongation via dCas9 binding has been shown to work in a variety of different bacterial species (Peters *et al*, 2016, 2019; Rock *et al*, 2017), so we expect the elongation repression/derepression mechanism to be applicable to other species. Transferring this mechanism to eukaryotes is more difficult because dCas9 is only capable of modest elongation repression in eukaryotic systems (Gilbert *et al*, 2013; Qi *et al*, 2013). Repression in eukaryotic systems is often accomplished through the fusion of chromatin‐remodeling proteins to dCas9, which are capable of robust and reversible repression of transcription (Gilbert *et al*, 2013, 2014; Zalatan *et al*, 2015; Mandegar *et al*, 2016). Derepressing these systems could be accomplished by targeting a dCas9 lacking the fused repression domains to the same DNA location. This approach would require two different dCas9 species, which could be accomplished using dCas9 orthologs (Esvelt *et al*, 2013; Kim *et al*, 2019; Gasiunas *et al*, 2020) or by encoding repression domain recruitment within the sgRNA scaffold (Zalatan *et al*, 2015).

Various approaches have been developed to place native genes under synthetic regulatory control through the insertion of synthetic promoters (e.g., that respond to T7 RNAP) or other regulatory motifs (Warner *et al*, 2010; Wang *et al*, 2012; Na *et al*, 2013). CRISPRi has been rapidly adopted because it can exert regulatory control without having to mutate the target; however, it only imparts a single on/off signal. To this end, depression/derepression can be used to inactivate the effects of CRISPRi in the control of native genes (Nielsen & Voigt, 2014; Weinberg *et al*, 2017; Moser *et al*, 2018; Henningsen *et al*, 2020). For example, CRISPRi/a has been used to dynamically repress or activate enzymes to control carbon flux in metabolic engineering applications (Moser *et al*, 2018; Peng *et al*, 2018; Lu *et al*, 2019; Tian *et al*, 2019; Fontana *et al*, 2020b). Derepression allows for the disruption of a subset of the genes being influenced by an activating or repressing sgRNA; in effect, this would introduce cis‐regulatory logic into the native genes without needing to insert operators. In this work, we had to deal with the challenge of identifying or designing PAM sites so that the sgR and sgD binding sites are appropriately positioned to satisfy the distance constraints. This restricts their utility in the control of genome‐encoded genes, where the likelihood of PAM sites being appropriately positioned in a promoter is small or restricts where the regulation can occur within a gene. This is likely why the effect that we observed for the blockage and release of elongation is small. However, recent efforts to engineer dCas9 to not require a PAM site have made progress (Kleinstiver *et al*, 2015; Hu *et al*, 2018; Nishimasu *et al*, 2018; Walton *et al*, 2020; Collias & Beisel, 2021). Collectively, these advances will allow the programming of cis‐regulatory logic to be applied to any gene in the genome without having to insert or modify genomic DNA.

## Materials and Methods

### Strains, plasmids, media, and chemicals

*Escherichia coli* NEB 10‐beta (C3019I, New England BioLabs, Ipswich, MA, USA) was used for all routine cloning. All genetic circuit measurements were done using *E. coli* K‐12 MG1655 * [F‐ λ‐ ilvG‐ rfb‐50 rph‐1 Δ(araCBAD) Δ(LacI)] (Blattner *et al*, 1997; Nielsen & Voigt, 2014). Cells were grown in in MOPS EZ Rich Defined Medium (Teknova, M2105) with 0.2% glucose (Teknova, G0520). Kanamycin (50 µg/ml, GoldBio, K‐120‐5) was used to maintain plasmids. Chemical inducers used the following: vanillic acid (Van) (Millipore Sigma, 94770); isopropyl β‐d‐1‐thiogalactopyranoside (IPTG) (GoldBio, I2481C); anhydrotetracycline (aTc) (Millipore Sigma, 37919); and choline chloride (Chol) (Millipore Sigma, C7017). DNA oligos and genes were ordered from Integrated DNA Technologies (Coralville, IA) and Twist Biosciences (San Francisco, CA). All plasmids were constructed from the parental pDAA038 backbone (Appendix Table S3) using TypeIIS assembly to insert circuit components between BsaI sites. A table of genetic parts and full plasmid sequences are provided in Appendix Table S2 and S3. Key plasmid maps are shown in Appendix Fig S5.

### Computational methods

The random 100‐bp sequence within P_C1_ was generated using the online Random DNA Sequence Generator (http://www.faculty.ucr.edu/˜mmaduro/random.htm) with the GC content set to 50%. Non‐linear fitting was completed with the Python scipy.optimize.curve_fit() function.

### Induction assays

All growth was performed in 96‐well V‐bottom plates (Roskilde, Denmark, #249952) at 1,000 rpm in a microplate shaker (ELMI, #DTS‐4). The day before, individual colonies were inoculated into 150 µl EZ Rich media and Kan for overnight (16 h) growth at 37°C. The next day, cultures were diluted 200‐fold by adding 0.75 µl of overnight culture into 150 µl of EZ Rich media and Kan. After 2‐h growth, cultures were diluted 1,000‐fold into inducing conditions by adding 6 µL of culture into 198 µl fresh media and Kan and then 5 µl of that dilution into media with inducers and Kan. To induce dCas9, aTc was added to a final concentration of 1.25 nM in all experiments. Growth was performed for 5.5 h, after which samples were prepared for flow cytometry.

### Flow cytometry analysis

Fluorescence characterization was performed using a BD LSR Fortessa flow cytometer with the HTS attachment (BD, Franklin Lakes, NJ). Samples were prepared by aliquoting 40 μl of cell culture into 160 μl of PBS containing 200 µg/ml Kan. All samples were run in standard mode at a flow rate of 2 μl/s. RFP fluorescence measurements were made using the green (561 nm) laser, and all data were derived from the PE‐Texas Red‐A channel (PMT voltage of 700 V). The FSC and SSC voltages were 650 and 270 V, respectively. At least 10,000 events were collected for each sample, and the Cytoflow Python package (https://github.com/cytoflow/cytoflow) was used for analysis, including gating. The geometric mean fluorescence is calculated for all cytometry distributions.

### Promoter input calculations

The following procedure was followed to convert inducer concentrations (e.g., [IPTG]) to the activities of the inducible promoter (e.g., P_Tac_) reported in RFP fluorescence (au). This approach has been described previously (Nielsen *et al*, 2016; Zhang & Voigt, 2018). Using a separate plasmid based on pDAA038, the response function of the inducible system was measured separately by measuring RFP fluorescence using cytometry. The RFP fluorescence values in au for the inducer concentrations were then plotted as “Promoter Activity”. The response functions used are shown in Appendix Fig S1.

### Repression/derepression dynamics

All growth was performed at 37°C and 350 rpm in an Innova44 (Eppendorf, NY, USA) with a 1‐inch throw. The day before the experiment, a starter culture was initiated by inoculating a colony into 2 ml EZ‐rich media with Kan in a 15‐ml test tube. The next day, this culture was diluted to an OD_600_ of 0.025 into 40 ml of fresh EZ‐rich media with Kan. This was allowed to grow for 2 h in a 250‐ml Erlenmeyer flask to bring the cells to OD_600_ = 0.5. At this point, a time point was taken for *t* = 0 and cells were diluted to OD_600_ = 0.00035 in an Erlenmeyer flask using fresh media with 10 mM Choline and 1.25 nM aTc. After 2 h of growth, 30‐min time points were taken. At each time point, 1 ml of culture was removed, spun down (1 min at 16,000 *g*), and then resuspended in 300 µl of PBS and 2 mg/ml Kan and analyzed by cytometry. To transfer into de‐repressing conditions, 28 ml of culture was washed twice by centrifugation at 12°C at 4,300 *g* for 5 min. Then, the cultures were diluted to OD_600_ = 0.035 in 40 ml of media with 100 µM Van, 10 mM Choline, and 1.25 nM aTc.

## Author contributions

DAA and CAV conceived the study and designed the experiments. DAA performed the experiments and analyzed the data. DAA and CAV wrote the manuscript.

## Conflict of interest

The authors declare that they have no conflict of interest.

## Supporting information



Appendix

## Data Availability

This study includes no data deposited in external repositories.
