# Supplementary material for: Competitive dCas9 binding as a mechanism for transcriptional control
Source: Mol Syst Biol. 2021 Nov 8;17(11):e10512. doi: 10.15252/msb.202110512 (PMC8574044; doi:10.15252/msb.202110512)
Supplement: Supplementary file 2 [file MSB-17-e10512-s002.pdf]

molecular systems biology

Competitive dCas9 binding as a mechanism for transcriptional control

Daniel Anderson and Christopher Voigt
DOI: 10.15252/msb.202110512

Corresponding author(s): Christopher Voigt (cavoigt@gmail.com)

Review Timeline:

Submission Date:	17th Jun 21
Editorial Decision:	8th Jul 21
Revision Received:	23rd Sep 21
Editorial Decision:	6th Oct 21
Revision Received:	10th Oct 21
Accepted:	11th Oct 21

Editor: Maria Polychronidou

Transaction Report:

(Note: With the exception of the correction of typographical or spelling errors that could be a source of ambiguity, letters and reports are not edited. Depending on transfer agreements, referee reports obtained elsewhere may or may not be included in this compilation. Referee reports are anonymous unless the Referee chooses to sign their reports.)

Thank you again for submitting your work to Molecular Systems Biology. We have now heard back from the three referees who agreed to evaluate your study. Overall, the reviewers acknowledge that the study is a relevant contribution to the field. They raise however a series of concerns, which we would ask you to address in a revision.

I think that the reviewers' recommendations are clear and it is therefore not required to repeat the points listed below. All issues raised by the reviewers would need to be satisfactorily addressed. Please contact me in case you would like to discuss in further detail any of the issues raised.

Reviewer #1:

In this study Anderson and Voigt, implement a CRISPR (via dCAS9) strategy in E. coli using pairs of sgRNAs designed to repress and then derepress transcription through competitive binding. Mechanistically, transcription is blocked when a repressing sgRNA (sgR) directs dCas9 to the first position and a second derepressing sgRNA (sgD) directs dCas9 to a mutually-exclusive second position that does not impact transcription. Notably, the sgD recruits dCas9 to an upstream site that blocks its ability to bind to the repressing position. In summary when this tool targets a promoter the authors achieve a 31-fold repression of RFP expression and a complete reactivation of the gene (i.e., de-repression) via the binding of an sgD transcript. However, the tool is less effective when targeting the reading frame. Namely, elongation control achieved 7-fold repression and

4-fold de-repression. In general, this is an interesting and relatively straightforward study that is well written, composed of high-quality and clearly presented data.

Minor Comments:

Comment 1.
In the introduction the authors correctly state: "As a design principle, combining operators is useful for "compressing" large logic operations to reduce the resource burden [33]." In addition to reference 33 (Rondon et al. Nature Communications, vol. 10, p. 4784, Oct 21 2019) the authors should also cite reference 13 (Groseclose et al. Nature Communications, vol. 11, pp. 1-15, 2020).

Comment 2.
I am assuming that Figure 2g is where the authors are basing the "31-fold repression and complete derepression" values. Accordingly it would be useful to mark the output values of RFP at 0 and 9mM Chol on the plot (i.e., where sgRP2 is not produced (0 Chol), and produced (9 Chol) - without the production of sgDP5). NOTE. Figure 2h is great, but this simple illustration would aid with the interpretation of the two input plot.

Comment 3.
In general a simple table with the measured values and corresponding bar graph of the key dynamic ranges articulated in the abstract (i.e., 34 and 7) in the supplement would be greatly appreciated.

Reviewer #2:

Summary: The authors describe the design and characterization of CRISPRi-based method for transcriptional regulation. The authors explored the design rules for creating a system employing two dCas9 ribonucleoproteins, one that represses the expression construct and one that derepressed the construct by displacing the first dCas9. The work characterizes the design rules necessary to achieve the derepression behavior as well as the fold activation that can be achieved from targeting transcription initiation as well as elongation. Lastly, the transcription initiation-targeting design was found to act in a radiometric manner.

General Remarks: The authors have implemented and characterized an approach that is a natural progression of CRISPRi technology but has nonobvious hurdles that have likely prevented previous implementation by others. The case presented for the two targeting methods is convincing and scientifically sound. As is, the work is a solid characterization of the designs.

Major Concerns:

1) Application of these circuits to one or more of the use cases referred to in the discussion would be compelling and raise the impact.

Minor Concerns:
1) Please rephrase the claim "although dCas9 can be engineered to remove the PAM sequence requirements" to soften it. Although the works cited have greatly broadened the PAM requirement, a PAM-free Cas9 has yet to be engineered to my knowledge.
2) Please consider adding the location of the PAM in the overview Figure 1A for clarity.

Reviewer #3:

In this study, the authors develop a CRISPR-based method for repressing and derepressing a target gene in bacteria. A repressive action is generated by using a sgRNA to recruit dCas9 to a position in the promoter or gene body that blocks transcription initiation or elongation ("sgR"). The derepressive action is then generated by simultaneously recruiting dCas9 to a nearby site using a different sgRNA ("sgD"), thereby displacing the repressive dCas9 molecule. These experiments were carried out in E. coli, where the method was evaluated for its ability to repress and derepress a gene encoding a fluorescent protein (rfp). The authors demonstrate that simultaneous expression of sgR and sgD leads to very effective derepression of rfp, which implies that this method is potentially transferrable to other experimental systems.

General remarks
The study is well designed and the experiments are rigorously performed and analyzed. The conclusions are supported by the data. The study's key conclusion is that this method represents a potentially useful tool for the synthetic biology toolbox that could be used when striving to program complex behaviors in bacteria. The authors do not demonstrate the usefulness of this system for anything other than regulating rfp, so the scope of the study is limited. The advance is of a purely technical nature

and will mostly be interesting for other researchers working in this very field. For this reason, I think that the significance of the study should be viewed in the general context of the rapidly moving field of synthetic biology, where it is valuable to disseminate new tools and methods rapidly. I therefore support the publication of this manuscript in Molecular Systems Biology after minor experimental and textual additions.

Minor points:
1. As the authors write, the method developed in this study can be useful for flexibly turning genes on and off in bacteria. But just how flexibly can the target gene be switched on and off? For example, can rfp expression be turned up and down repeatedly by nimbly modulating sgR and sgD expression? For example, the data in Fig. 2g and h show a quite static picture of what happens at the steady state. But it would be useful if this method could be used to switch genes rapidly and repeatedly on and off. Currently, the authors show that sgD effectively derepresses the sgR-bound promoter when sgR and sgD expression is induced simultaneously. But can sgD derepress as effectively if its expression is induced after sgR expression, when sgR is already bound to the promoter? And conversely, can sgR dislodge sgD and repress the target gene if sgR is expressed after sgD? As the authors note, once dCas9 forms a ternary complex on the target DNA, it remains bound almost irreversibly. And in the Discussion, the authors mention that the competitive action between sgR and sgD may depend on which of the two binds DNA first. I agree that this sounds plausible. However, it would be powerful if this system could be used to turn target genes nimbly and repeatedly on and off, and it would therefore be important to know more about the kinetics of this system: For example, how are the repressive/derepressive actions affected if sgR and sgD are turned on one after the other rather than simultaneously? E. coli can divide every 20 minutes (displacing Cas9 from the rfp promoter) and that it may therefore not matter much in practice whether sgR or sgD is induced first, but still, it would be useful to know something about the kinetics of the system.

2. It is remarkable that sgD can reverse sgR repression of rfp to the extent that rfp expression returns all the way back to pre-sgR levels (Fig. 2g). sgD obviously blocks sgR binding very effectively, but I would still intuitively expect that sgR is in turn equally capable of blocking sgD binding. In other words, I would expect that the coexpression of sgR and sgD would result in competitive binding equilibrium at the rfp promoter such that there would always be at least some sgR bound at the promoter, preventing rfp levels to return completely back to pre-sgR levels. Instead, the data in Fig. 4 suggest that sgD is much more capable of displacing sgR than sgR is capable of displacing sgD, as this "sgD dominance" occurs even at equal expression levels of sgD and sgR. Is this a proper interpretation? If so, could you comment on this?

3. Fig. 4 would be easier to understand if (a) and (b) were labeled with "Promoter" and "Gene body", respectively.

4. The "bubble" mentioned on p. 3 is called an R-loop.

5. The scope of the study is focused on E. coli. It remains open whether a similar system could be used in eukaryotic cells; dCas9 binding may not be sufficient to block transcription initiation or elongation to the necessary degree. The authors could include a short paragraph in the Discussion reflecting on the potential of this system to be transferred to eukaryotes. I also think that the title of the study should reflect the focus of the study, for example "Competitive dCas9 binding as a mechanism for transcriptional control in bacteria"

6. The paper should expand their discussion how the system can be used in bacteria species other than E. coli. The authors should cite PMID: 27238023, as it provides related evidence that CRISPRi can be used robustly in other bacteria species.

Reviewer #1:

1. *In the introduction the authors correctly state: "As a design principle, combining operators is useful for "compressing" large logic operations to reduce the resource burden [33]." In addition to reference 33 (Rondon et al. Nature Communications, vol. 10, p. 4784, Oct 21 2019) the authors should also cite reference 13 (Groseclose et al. Nature Communications, vol. 11, pp. 1-15, 2020).*

   The citation has been added.

2. *I am assuming that Figure 2g is where the authors are basing the "31-fold repression and complete derepression" values. Accordingly, it would be useful to mark the output values of RFP at 0 and 9mM Chol on the plot (i.e., where sgRP2 is not produced (0 Chol), and produced (9 Chol) - without the production of sgDP5). NOTE: Figure 2h is great, but this simple illustration would aid with the interpretation of the two-input plot.*

   The figure edits have been made.

3. *In general, a simple table with the measured values and corresponding bar graph of the key dynamic ranges articulated in the abstract (i.e., 34 and 7) in the supplement would be greatly appreciated.*

   Sentences were added to appropriate figure captions to indicate where the fold-repression and fold-derepression dynamic ranges were derived. Promoter fold-repression and fold-derepression dynamic ranges are described in the Figure 2h caption. Elongation fold-repression and fold-derepression dynamic ranges are described in Figure 4h caption.

Christopher A. Voigt
Professor of Biological Engineering

Synthetic Biology Center
Dept of Biological Engineering

617.324.4851
cavoigt@gmail.com

Building NE47-140
500 Technology Square
Cambridge, Massachusetts 02139

Reviewer #2:

1. *Application of these circuits to one or more of the use cases referred to in the discussion would be compelling and raise the impact.*

We have not yet applied this to one of the use cases described.

2. *Please rephrase the claim "although dCas9 can be engineered to remove the PAM sequence requirements" to soften it. Although the works cited have greatly broadened the PAM requirement, a PAM-free Cas9 has yet to be engineered to my knowledge.*

The text has been edited as suggested.

3. *Please consider adding the location of the PAM in the overview Figure 1A for clarity.*

The figure has been edited as suggested.

Christopher A. Voigt
Professor of Biological Engineering
Synthetic Biology Center
Dept of Biological Engineering
617.324.4851
cavoigt@gmail.com
Building NE47-140
500 Technology Square
Cambridge, Massachusetts 02139

Reviewer #3:

1. As the authors write, the method developed in this study can be useful for flexibly turning genes on and off in bacteria. But just how flexibly can the target gene be switched on and off? For example, can rfp expression be turned up and down repeatedly by nimbly modulating sgR and sgD expression? For example, the data in Fig. 2g and h show a quite static picture of what happens at the steady state. But it would be useful if this method could be used to switch genes rapidly and repeatedly on and off. Currently, the authors show that sgD effectively derepresses the sgR-bound promoter when sgR and sgD expression is induced simultaneously. But can sgD derepress as effectively if its expression is induced after sgR expression, when sgR is already bound to the promoter? And conversely, can sgR dislodge sgD and repress the target gene if sgR is expressed after sgD? As the authors note, once dCas9 forms a ternary complex on the target DNA, it remains bound almost irreversibly. And in the Discussion, the authors mention that the competitive action between sgR and sgD may depend on which of the two binds DNA first. I agree that this sounds plausible. However, it would be powerful if this system could be used to turn target genes nimbly and repeatedly on and off, and it would therefore be important to know more about the kinetics of this system: For example, how are the repressive/derepressive actions affected if sgR and sgD are turned on one after the other rather than simultaneously? E. coli can divide every 20 minutes (displacing Cas9 from the rfp promoter) and that it may therefore not matter much in practice whether sgR or sgD is induced first, but still, it would be useful to know something about the kinetics of the system.

   As suggested, dynamic experiments have been added to make a new Figure 3.

2. *It is remarkable that sgD can reverse sgR repression of rfp to the extent that rfp expression returns all the way back to pre-sgR levels (Fig. 2g). sgD obviously blocks sgR binding very effectively, but I would still intuitively expect that sgR is, in turn, equally capable of blocking sgD binding. In other words, I would expect that the coexpression of sgR and sgD would result in competitive binding equilibrium at the rfp promoter such that there would always be at least some sgR bound at the promoter, preventing rfp levels to return completely back to pre-sgR levels. Instead, the data in Fig. 4 suggest that sgD is much more capable of displacing sgR than sgR is capable of displacing sgD, as this "sgD dominance" occurs even at equal expression levels of sgD and sgR. Is this a proper interpretation? If so, could you comment on this?*

   This asymmetry may be due to sequence-dependent binding strength differences. Different sgRNA sequences have been shown to have different apparent binding strengths for their target sequences. In fact, the binding constants derived from model fits for sgR_{P2} and sgD_{P5} indicate that sgD is 1.5-fold tighter binding than sgR (Table 1). Additionally, if sgD and sgR are both occupying the promoter with equal probability (sgD / sgR ratio = 1), we would only expect to observe a 2-fold reduction from maximum expression levels. Since the y-axis of Figure 5a,b are logarithmically-scaled, a two-fold change would appear relatively insignificant.

3. *Fig. 4 would be easier to understand if (a) and (b) were labeled with "Promoter" and "Gene body",*

Christopher A. Voigt
Professor of Biological Engineering

Synthetic Biology Center
Dept of Biological Engineering

617.324.4851
cavoigt@gmail.com

Building NE47-140
500 Technology Square
Cambridge, Massachusetts 02139

respectively.

The figure has been edited appropriately.

4. *The "bubble" mentioned on p. 3 is called an R-loop.*

The text has been edited.

5. *The scope of the study is focused on E. coli. It remains open whether a similar system could be used in eukaryotic cells; dCas9 binding may not be sufficient to block transcription initiation or elongation to the necessary degree. The authors could include a short paragraph in the Discussion reflecting on the potential of this system to be transferred to eukaryotes. I also think that the title of the study should reflect the focus of the study, for example "Competitive dCas9 binding as a mechanism for transcriptional control in bacteria"*

A paragraph has been added to the discussion.

6. *The paper should expand their discussion how the system can be used in bacteria species other than E. coli. The authors should cite PMID: 27238023, as it provides related evidence that CRISPRi can be used robustly in other bacteria species.*

The citation has been added.

Thank you again for submitting your work to Molecular Systems Biology. We have now heard back from the reviewer who was asked to evaluate the study. The reviewer is satisfied with the modifications made and is supportive of publication.

Before we formally accept the study for publication, we would ask you to perform some minor edits.

Reviewer #2:

The authors have addressed my concerns. This is a great paper and I support publication.

The authors have made all requested editorial changes.

Thank you again for sending us your revised manuscript. We are now satisfied with the modifications made and I am pleased to inform you that your paper has been accepted for publication.

EMBO PRESS

YOU MUST COMPLETE ALL CELLS WITH A PINK BACKGROUND ➔

PLEASE NOTE THAT THIS CHECKLIST WILL BE PUBLISHED ALONGSIDE YOUR PAPER

Corresponding Author Name: Christopher A. Voigt
Journal Submitted to: Molecular Systems Biology
Manuscript Number: MSB-2021-10512

USEFUL LINKS FOR COMPLETING THIS FORM

http://www.antibodypedia.com
http://1degreebio.org
http://www.equator-network.org/reporting-guidelines/improving-bioscience-research-repor
http://grants.nih.gov/grants/olaw/olaw.htm
http://www.mrc.ac.uk/Ourresearch/Ethicsresearchguidance/Useofanimals/index.htm
http://ClinicalTrials.gov
http://www.consort-statement.org
http://www.consort-statement.org/checklists/view/32-consort/66-title
http://www.equator-network.org/reporting-guidelines/reporting-recommendations-for-tumo
http://datadryad.org
http://figshare.com
http://www.ncbi.nlm.nih.gov/gap
http://www.ebi.ac.uk/ega
http://biomodels.net/
http://biomodels.net/miriam/
http://jjj.biochem.sun.ac.za
https://osp.od.nih.gov/biosafety-biosecurity-and-emerging-biotechnology/
http://www.selectagents.gov/

Reporting Checklist For Life Sciences Articles (Rev. June 2017)

This checklist is used to ensure good reporting standards and to improve the reproducibility of published results. These guidelines are consistent with the Principles and Guidelines for Reporting Preclinical Research issued by the NIH in 2014. Please follow the journal's authorship guidelines in preparing your manuscript.

A- Figures
1. Data
The data shown in figures should satisfy the following conditions:

- ➔ the data were obtained and processed according to the field's best practice and are presented to reflect the results of the experiments in an accurate and unbiased manner.
- ➔ figure panels include only data points, measurements or observations that can be compared to each other in a scientifically meaningful way.
- ➔ graphs include clearly labeled error bars for independent experiments and sample sizes. Unless justified, error bars should not be shown for technical replicates.
- ➔ if n< 5, the individual data points from each experiment should be plotted and any statistical test employed should be justified
- ➔ Source Data should be included to report the data underlying graphs. Please follow the guidelines set out in the author ship guidelines on Data Presentation.

2. Captions

Each figure caption should contain the following information, for each panel where they are relevant:

- ➔ a specification of the experimental system investigated (eg cell line, species name).
- ➔ the assay(s) and method(s) used to carry out the reported observations and measurements
- ➔ an explicit mention of the biological and chemical entity(ies) that are being measured.
- ➔ an explicit mention of the biological and chemical entity(ies) that are altered/varied/perturbed in a controlled manner.

- ➔ the exact sample size (n) for each experimental group/condition, given as a number, not a range;
- ➔ a description of the sample collection allowing the reader to understand whether the samples represent technical or biological replicates (including how many animals, litters, cultures, etc.).
- ➔ a statement of how many times the experiment shown was independently replicated in the laboratory.
- ➔ definitions of statistical methods and measures:
  - common tests, such as t-test (please specify whether paired vs. unpaired), simple χ2 tests, Wilcoxon and Mann-Whitney tests, can be unambiguously identified by name only, but more complex techniques should be described in the methods section;
  - are tests one-sided or two-sided?
  - are there adjustments for multiple comparisons?
  - exact statistical test results, e.g., P values = x but not P values < x;
  - definition of 'center values' as median or average;
  - definition of error bars as s.d. or s.e.m.

Any descriptions too long for the figure legend should be included in the methods section and/or with the source data.

In the pink boxes below, please ensure that the answers to the following questions are reported in the manuscript itself. Every question should be answered. If the question is not relevant to your research, please write NA (non applicable). We encourage you to include a specific subsection in the methods section for statistics, reagents, animal models and human subjects.

B- Statistics and general methods

Please fill out these boxes ➔ (Do not worry if you cannot see all your text once you press return)

1.a. How was the sample size chosen to ensure adequate power to detect a pre-specified effect size?	Effect size was not pre-specified. At least three replicates were completed for each data point.
1.b. For animal studies, include a statement about sample size estimate even if no statistical methods were used.	NA
2. Describe inclusion/exclusion criteria if samples or animals were excluded from the analysis. Were the criteria pre-established?	All samples were included.
3. Were any steps taken to minimize the effects of subjective bias when allocating animals/samples to treatment (e.g. randomization procedure)? If yes, please describe.	NA
For animal studies, include a statement about randomization even if no randomization was used.	NA
4.a. Were any steps taken to minimize the effects of subjective bias during group allocation or/and when assessing results (e.g. blinding of the investigator)? If yes please describe.	NA
4.b. For animal studies, include a statement about blinding even if no blinding was done	NA
5. For every figure, are statistical tests justified as appropriate?	NA
Do the data meet the assumptions of the tests (e.g., normal distribution)? Describe any methods used to assess it.	NA
Is there an estimate of variation within each group of data?	NA

Is the variance similar between the groups that are being statistically compared?	NA

C- Reagents

6. To show that antibodies were profiled for use in the system under study (assay and species), provide a citation, catalog number and/or clone number, supplementary information or reference to an antibody validation profile. e.g., Antibodypedia (see link list at top right), 1DegreeBio (see link list at top right).	NA
7. Identify the source of cell lines and report if they were recently authenticated (e.g., by STR profiling) and tested for mycoplasma contamination.	NA

* for all hyperlinks, please see the table at the top right of the document

D- Animal Models

8. Report species, strain, gender, age of animals and genetic modification status where applicable. Please detail housing and husbandry conditions and the source of animals.	NA
9. For experiments involving live vertebrates, include a statement of compliance with ethical regulations and identify the committee(s) approving the experiments.	NA
10. We recommend consulting the ARRIVE guidelines (see link list at top right) (PLoS Biol. 8(6), e1000412, 2010) to ensure that other relevant aspects of animal studies are adequately reported. See author guidelines, under 'Reporting Guidelines'. See also: NIH (see link list at top right) and MRC (see link list at top right) recommendations. Please confirm compliance.	NA

E- Human Subjects

11. Identify the committee(s) approving the study protocol.	NA
12. Include a statement confirming that informed consent was obtained from all subjects and that the experiments conformed to the principles set out in the WMA Declaration of Helsinki and the Department of Health and Human Services Belmont Report.	NA
13. For publication of patient photos, include a statement confirming that consent to publish was obtained.	NA
14. Report any restrictions on the availability (and/or on the use) of human data or samples.	NA
15. Report the clinical trial registration number (at ClinicalTrials.gov or equivalent), where applicable.	NA
16. For phase II and III randomized controlled trials, please refer to the CONSORT flow diagram (see link list at top right) and submit the CONSORT checklist (see link list at top right) with your submission. See author guidelines, under 'Reporting Guidelines'. Please confirm you have submitted this list.	NA
17. For tumor marker prognostic studies, we recommend that you follow the REMARK reporting guidelines (see link list at top right). See author guidelines, under 'Reporting Guidelines'. Please confirm you have followed these guidelines.	NA

F- Data Accessibility

18: Provide a "Data Availability" section at the end of the Materials & Methods, listing the accession codes for data generated in this study and deposited in a public database (e.g. RNA-Seq data: Gene Expression Omnibus GSE39462, Proteomics data: PRIDE PXD000208 etc.) Please refer to our author guidelines for 'Data Deposition'.<br><br>Data deposition in a public repository is mandatory for:<br>a. Protein, DNA and RNA sequences<br>b. Macromolecular structures<br>c. Crystallographic data for small molecules<br>d. Functional genomics data<br>e. Proteomics and molecular interactions	Done. DNA sequences are available in the Appendix.
19. Deposition is strongly recommended for any datasets that are central and integral to the study; please consider the journal's data policy. If no structured public repository exists for a given data type, we encourage the provision of datasets in the manuscript as a Supplementary Document (see author guidelines under 'Expanded View' or in unstructured repositories such as Dryad (see link list at top right) or Figshare (see link list at top right).	DNA sequences are available in the Appendix.
20. Access to human clinical and genomic datasets should be provided with as few restrictions as possible while respecting ethical obligations to the patients and relevant medical and legal issues. If practically possible and compatible with the individual consent agreement used in the study, such data should be deposited in one of the major public access-controlled repositories such as dbGAP (see link list at top right) or EGA (see link list at top right).	NA
21. Computational models that are central and integral to a study should be shared without restrictions and provided in a machine-readable form. The relevant accession numbers or links should be provided. When possible, standardized format (SBML, CellML) should be used instead of scripts (e.g. MATLAB). Authors are strongly encouraged to follow the MIRIAM guidelines (see link list at top right) and deposit their model in a public database such as Biomodels (see link list at top right) or JWS Online (see link list at top right). If computer source code is provided with the paper, it should be deposited in a public repository or included in supplementary information.	NA

G- Dual use research of concern

22. Could your study fall under dual use research restrictions? Please check biosecurity documents (see link list at top right) and list of select agents and toxins (APHIS/CDC) (see link list at top right). According to our biosecurity guidelines, provide a statement only if it could.	No.
